# Predicting survival in patients with acute decompensated heart failure complicated by cardiogenic shock

**DOI:** 10.1016/j.ijcha.2021.100809

**Published:** 2021-06-04

**Authors:** Nuccia Morici, Giovanna Viola, Laura Antolini, Gianfranco Alicandro, Michela Dal Martello, Alice Sacco, Maurizio Bottiroli, Federico Pappalardo, Luca Villanova, Laura De Ponti, Carlo La Vecchia, Maria Frigerio, Fabrizio Oliva, Justin Fried, Paolo Colombo, Arthur Reshad Garan

**Affiliations:** a Unità di Cure Intensive Cardiologiche and De Gasperis Cardio-Center, ASST Grande Ospedale Metropolitano Niguarda, Milan, Italy; b Department of Clinical Sciences and Community Health, Università degli Studi di Milano, Milan, Italy; c School of Medicine, Center of Bioststistics for Clinical Epidemiology, Univ. Milano Bicocca, Monza, Italy; d Cardio-thoracic Intensive Care Unit and De Gasperis Cardio-Center, ASST Grande Ospedale Metropolitano Niguarda, Milan, Italy; e Dept. Anesthesia and Intensive Care, IRCCS ISMETT, UPMC Italy, Palermo; f Heart Failure and Cardiac Transplant Unit, ASST Grande Ospedale Metropolitano Niguarda, Milan, Italy; g Division of Cardiology, Department of Medicine, Columbia University Medical Center–New York Presbyterian, NewYork, NewYork, USA; h Division of Cardiology, Department of Medicine, Beth Israel Deaconess Medical Center, Harvard Medical School, Boston, MA, USA

**Keywords:** Cardiogenic shock, Prognostication, Net benefit, Acute decompensated heart failure, Heart replacement therapy, Heart failure

## Abstract

**Background:**

Acute decompensated heart failure (ADHF) complicated by cardiogenic shock (CS) has unique pathophysiological background requiring specific patient stratification, management and therapeutic targets. Accordingly, the aim of this study was to derive a simple stratification tool to predict survival in patients with ADHF complicated by CS.

**Methods and results:**

Using logistic regression, univariable testing was performed to identify the variables potentially associated with 28-day mortality. We propose a new logistic model (ALC-Shock score) based on three easy parameters (age, serum creatinine and serum lactate at the ICU admission) as a powerful predictor of survival or successful bridge to heart replacement therapy at 28-day follow-up in this specific population. A multivariable analysis (logistic model) was performed to evaluate the association between selected variables and outcome (overall death at 28-day follow up). The score was then validated in a different cohort of 93 ADHF-CS patients and compared to a previous developed score (the Cardshock score).

Overall, 28-day mortality was 34%. The ALC-shock score showed better discrimination (Area Under the Curve-AUC- 0.82; 95% CI 0.73–0.91) as compared to the Cardshock score (AUC 0.67; 95% CI 0.55–0.79) (p = 0.009) to predict 28-days overall mortality. In the validation cohort the AUC for the ALC-shock score was 0.66.

**Conclusions:**

A simple score including age, lactates and creatinine on admission could be considered to predict short-term mortality in CS-ADHF patients in order to drive towards a treatment intensification.

## 1 Introduction

Cardiogenic shock (CS) is a clinical challenge which results from complex and distinct pathways leading to oxygen starvation [1], [2], [3]. Despite improvements in hemodynamics with short-term mechanical support and advances in intensive care unit (ICU) management, in-hospital mortality for CS patients remains as high as 50%, and stagnant over time [4], [5].

Data from American and European registries have recently highlighted a rising prevalence of CS related to acute decompensated heart failure (ADHF) as opposed to acute myocardial infarction (AMI) [6]. Since the underlying pathologies are different, [6] there is an unmet need to identify disease-specific, dedicated risk scores that are readily available upon ICU admission, which might ultimately guide choice of therapies, and thereby improve outcomes and optimize resource allocation.

However, data on patients with CS not related to AMI are lacking even in large, well-reported registries [7], [8], [9], [10], [11], [12], [13] and are mostly limited to case-series, [14], [15], [16], [17], [18], [19], [20], [21], [22] with only one phase II study [23] and one small randomized clinical trial [24]. In addition, only one risk score has been recently validated in a cohort of patients that included a significant number of ADHF-CS patients [25]. However, this score was based not only on clinical and laboratory variables, but also on hemodynamic criteria that may not be consistently available upon admission to the ICU [25].

Thus, the aim of this study was to derive a simple score that could predict 28-day mortality of ADHF-CS patients based on variables that are obtained easily and clinically relevant, and to compare it with the CardShock score, [13] which has shown good results in predicting short-term mortality in CS related to a large spectrum of etiologies, but, mainly, to acute coronary syndrome.

## 2 Methods

### 2.1 Study design

We carried out a retrospective cohort study by reviewing medical charts of two groups of ADHF-CS patients: the first group (derivation cohort) was used to develop a prediction model for 28-day overall mortality that was then validated in the second group (validation cohort).

The derivation cohort included 87 consecutive ADHF-CS patients admitted between 2015 and 2019 at Intensive Coronary Care Unit (ICCU) and Cardio-thoracic Intensive Care Unit (CICU) of ASST Grande Ospedale Metropolitano Niguarda within 12 h of CS diagnosis.

The validation cohort included 93 ADHF-CS patients, admitted between January 2011 and April 2016, who received and intra-aortic balloon pump (IABP) for hemodynamic support in the setting of CS in the ICU of the Columbia University Irving Medical Center, New York, NY.

Patients were included if the met all three following criteria: 1) were aged 18–74; 2) had systolic blood pressure (SBP) < 90 mmHg or mean arterial pressure (MAP) < 60 mmHg (after an appropriate fluid challenge if there were no sign of overt fluid overload), or need of vasoactive agents to maintain SBP > 90 mmHg or MAP > 60 mmHg); 3) had at least one of the following evidence of severe hypoperfusion: altered state of consciousness; sweaty and cold skin; mixed venous oxygen saturation < 60%; arterial lactates > 2 mmol/L; oliguria < 0.5 ml/Kg/h for at least 6 h.

Patients were excluded if any of the following criteria were present: 1) CS symptoms beyond 12 h; 2) septic shock with identified infection; 3) CS due to AMI defined according to current guidelines [26]; 4) CS due to acute myocarditis; 5) CS due to pulmonary thromboembolism; 6) constrictive pericarditis; 6) congenital heart disease; 7) CS secondary to either cardiac or non-cardiac surgery.

The study was conducted in accordance with ethical principles based on Helsinki’s Declaration, [27] International Conference on Harmonization for Good Clinical Practice, and the current ethical rules. The Strengthening the Reporting of Observational Studies in Epidemiology Guidelines (STROBE) were followed for reporting the findings [28]. This study was approved by the Local Ethics Committee of Milano Area 3 of the ASST Grande Ospedale Metropolitano Niguarda (Piazza Ospedale Maggiore 3, 20,162 Milano) and by the Institutional Review Board of the Columbia University Medical Center. As a primarily quality improvement effort collecting and reporting observational data relating to patients presenting with CS, data were collected using existing medical record data and the appropriate bodies at each center waived the requirement of written consent.

### 2.2 Data analysis

Baseline characteristics were compared between patients alive and deceased at 28 days. Continuous data are presented as mean ± standard deviation (SD) or median (interquartile range, IQR) and were compared between groups using the Student’s *t* test or Mann-Whitney test, as appropriate. Categorical variables were compared between groups using the Χ^2^ test.

Using logistic regression, univariable testing was performed to identify risk factors for 28-day mortality among the following variables at baseline: age, gender, body mass index, systolic blood pressure, heart rate, laboratory examination (bilirubin, creatinine and international normalized ratio, hemoglobin, central venous pressure and lactate on admission (Table 1). To avoid overfitting, we decided a priori to include only a limited number of clinically relevant variables in the multivariable logistic model and to compute probability of 28-day mortality. Different models, including 5 outcome events for predictor variable, were tested, leading to the final model which includes only three predictors: age, lactates on admission and serum creatinine.

**Table 1:** Demographic, clinical, biochemical characteristics upon admission and non-pharmacologic support during hospitalization in patients included in the derivation cohort.

	Overall population (n = 87)	Patients who survived (n = 57)	Patients who died (n = 30)	p value
Age, years	50.8 ± 15.2	46.7 ± 14.9	58.8 ± 12.2	<0.001
Male sex	64 (73.5)	41 (71.9)	23 (76.7)	0.634
BMI	24.0 ± 4.4	23.9 ± 4.4	24.2 ± 4.4	0.842
History of Diabetes mellitus	18 (20.7)	12 (21.0)	6 (20.0)	0.908
History of Hypertension	22 (25.3)	10 (17.5)	12 (40.0)	0.022
History of Dyslipidemia	20 (22.9)	11 (19.3)	9 (30.0)	0.259
Preexisting CAD	24 (27.6)	12 (21.0)	12 (40.0)	0.060
Etiology Hypokinetic cardiomyopathy Post-ischemic cardiomyopathy Valvular cardiomyopathy Hypertrophic cardiomyopathy Post-chemotherapy Other	38 (43.2) 23 (26.1) 10 (11.4) 5 (5.7) 5 (5.7) 6 (6.9)	29 (50.0) 11 (19) 5 (8.6) 3 (5.2) 5 (8.6) 4 (7.0)	9 (30.0) 12 (40) 5 (16.7) 2 (6.7) 0 2 (6.6)	0.154
LVEF	21.3 ± 9.6	20.7 ± 9.7	22.6 ± 9.6	0.398
History of CRF	32 (36.8)	13 (22.8)	19 (63.3)	<0.001
Heart rate	94.5 ± 19.7	95.6 ± 19.3	92.4 ± 20.6	0.477
SAP, mmHg	91 ± 15.1	95.5 ± 12.4	83.6 ± 16.8	<0.001
DAP, mmHg	54 ± 14.2	56.6 ± 14.5	49.8 ± 12.8	0.039
MAP, mmHg	67 ± 13	69.4 ± 12.6	61.2 ± 12.4	0.005
Wedge pressure, mmHg	13.8 ± 8.0	12.0 ± 6.9	17.5 ± 9.2	0.138
CVP, mmHg	12.2 ± 6.8	11.1 ± 6.2	14.6 ± 7.6	0.034
mPAP, mmHg	23.7 ± 10.1	20.7 ± 6.8	30.3 ± 13.5	0.052
SVcO2	55 ± 14.3	55.2 ± 14.4	53.6 ± 14.3	0.689
Arterial lactates, mmol/L	3.8 ± 3.5	3.1 ± 2.7	5.2 ± 4.4	0.008
Serum creatinine, mg/dl	1.76 ± 1.1	1.4 ± 0.7	2.3 ± 1.3	<0.001
Serum bilirubin, mg/dl	1.98 ± 1.7	1.7 ± 1.5	2.4 ± 2.0	0.109
INR	2.2 ± 1.2	2.1 ± 1.1	2.4 ± 1.4	0.206
Hemoglobin, gr/dl	12.2 ± 1.9	12.4 ± 2.0	11.9 ± 1.6	0.223
Platelet count, x10^9^/L	234 ± 93	241 ± 96	219 ± 87	0.288
Troponin T HS, ng/L	86 (39–231)	59 (33–139)	140 (56–700)	0.029
Diuresis, ml/Kg/h	1 ± 0.8	0.7 ± 0.9	0.5 ± 0.6	0.235
RRT	6 (6.9)	4 (7.0)	2 (6.7)	0.969
Inotropic score	11 (7–20)	9 (6–15)	19 (12–25)	<0.001
Mechanical ventilation	40 (45.9)	24 (42.1)	16 (53.3)	0.157
NIMV	36 (41.4)	21 (36.8)	15 (50)	0.487
IABP*	62 (71.3)	45 (78.9)	17 (56.7)	0.029
ECMO^	22 (25.9)	14 (24.6)	8 (26.7)	0.830
Time to LAVD, days	7 (1–24)	3 (0–27)	15 (10–21)	0.448
Time to Heart Transplantation	14 (7–21)	14 (7–34)	3 (0–14)	0.102

Data are reported as mean and standard deviation or number and percentage.

BMI: Body Mass Index; CRF: Chronic Renal Failure; CVP: Central Venous Pressure; RRT: Renal Replacement Therapy; DAP: Diastolic Arterial Pressure; ECMO: ExtraCorporeal Membrane Oxigenation; IABP: IntraAortic Ballon Pump; LVAD: left ventricular assist device: LVEF: Left Ventricle Ejection Fraction; MAP: Mean Arterial Pressure; mPAP: mean Pulmonary Artery Pressure; NIMV: Non-Invasive Mechanical Ventilation; SAP: Systolic Arterial Pressure.

*1 patient had missing data

^time to ECMO ha a mean of 2 days (SD 1) for patients who survived and 9 days (SD 6) for patients who died.

Only 33 patients received pulmonary artery catheter for invasive hemodynamic monitoring at ICU admission.

We compared the performance of our prognostic score with that of the CardShock score [13] in the derivation cohort, though this was not performed in the validation cohort because necessary data for calculating this score were not available for this cohort. For both score, discrimination was assessed with c-statistics, whereas calibration was assessed using the risk categories defined by tertiles of individual predicted probabilities of 28-day mortality.

The clinical usefulness of the two scores was evaluated by estimating the net benefit of using the model to risk-stratify patients according to different decision thresholds of 28-day mortality [29].

As a sensitivity analysis, since the validation cohort included only patients who were treated with IABP, we compared observed and predicted 28-day mortality between the validation cohort and the subgroup of the derivation cohort which also received IABP support (62 patients) according to tertiles of predicted probabilities.

Considering the main aim of our study to identify baseline predictors of 28-day mortality, we did not include neither short- nor long-term mechanical support, nor heart transplantation as predictors or endpoints, since they are key elements of optimal treatment for end-stage HF patients.

All analyses were performed using STATA version 14 (Stata Corp., College Station, TX) and R software version 3.5.1.

## 3 Results

### 3.1 Clinical characteristics of the derivation cohort

Three-hundred and forty patients were identified and screened from medical records; 253 patients were excluded, and 87 patients were included. Demographic, clinical, and biochemical characteristics upon admission and non-pharmacologic support during hospitalization of the study population are reported in Table 1 according to mortality at 28 days.

**Fig. 1:**
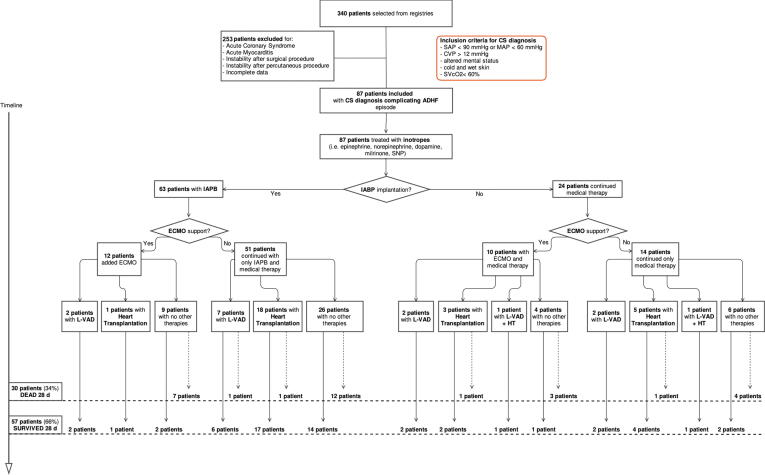
Title: Patients’ flow. Caption: Description of inclusion and exclusion criteria and detailed therapeutic management for the derivation cohort. Among IABP patients (63), 1 had missing data for relevant covariates.

The most frequently reported etiology was non-ischemic dilated cardiomyopathy, followed by ischemic dilated cardiomyopathy. In this derivation cohort, 30 patients (34%) had expired at 28 days and 57 (66%) were alive; among them, 19 (33%) experienced improvement and were discharged on medical therapy and 38 (67%) were bridged to heart replacement therapy [12 patients to left ventricle assist device (LVAD), 24 patients to heart transplantation (HT) and 2 patients to HT after LVAD] (Fig. 1). Patients who survived were younger, less frequently suffering from hypertension and chronic renal failure. Hypotension and increased serum creatinine, arterial lactates and central venous pressure upon admission were more frequent in patients who died. Among non-pharmacological support, IABP was more frequently implanted in patients who survived. In the derivation cohort only 33 patients received invasive monitoring with pulmonary artery catheter at ICU admission. Of note, as per institutional policy, pulmonary artery catheter insertion followed initiation of medical treatment with inotropes, vasodilators, intra-aortic balloon pump, thus potentially accounting for the lower wedge pressure readings at baseline.

### 3.2 Clinical characteristics of the validation cohort

The validation cohort included 93 patients with ADHF and hemodynamic evidence of CS who underwent IABP implantation. Baseline characteristics are reported in Table 2. Thirty patients (32%) died at 28-days follow up, whereas 63 (68%) survived, 21 discharged on medical therapy (33%) and 42 bridged to heart replacement therapy (67%) [4 patients to HT and 38 patients to LVAD].

**Table 2:** Demographic, clinical, biochemical characteristics upon admission of the patients included in the validation cohort.

	Overall population (n = 93)	Patients who survived (n = 63)	Patients who died (n = 30)	p value
Age, years	58.5 ± 13.8	56.0 ± 14.0	63.7 ± 12.2	0.011
Male sex	77 (82.8)	52 (82.5)	25 (83.3)	0.924
BMI	27.5 ± 7.3	27.1 ± 7.2	28.6 ± 7.8	0.522
Diabetes mellitus	30 (32.3)	15 (23.8)	15 (50.0)	0.012
Hypertension*	24 (46.1)	18 (47.4)	6 (42.9)	0.772
CRT*	21 (40.4)	16 (42.1)	5 (35.7)	0.677
Heart rate	98.0 ± 20.6	99.4 ± 19.4	95.1 ± 23.1	0.342
SAP, mmHg	100.2 ± 14.3	101.3 ± 15.4	97.8 ± 11.8	0.261
DAP, mmHg	64 ± 12.4	66.2 ± 12.3	59.4 ± 11.5	0.012
MAP, mmHg	76.1 ± 11.4	77.9 ± 11.8	72.2 ± 9.5	0.022
Wedge pressure, mmHg	30.2 ± 11.4	33.7 ± 10.3	23.9 ± 11.2	0.026
CVP, mmHg	17.2 ± 7.2	16.9 ± 7.2	17.8 ± 7.3	0.587
mPAP, mmHg	38.5 ± 10.6	40.0 ± 9.9	35.4 ± 11.4	0.049
SVcO2	41.3 ± 12.1	40.8 ± 12.6	43.0 ± 10.7	0.557
Arterial lactates, mmol/L	1.6 (1.2–2.5)	1.4 (1.1–2.2)	2.2 (1.4–8)	0.003
Serum creatinine, mg/dl	1.8 (1.4–2.5)	1.7 (1.4–2.6)	1.8 (1.5–2.4)	0.796
CPO	0.54 ± 0.16	0.53 ± 0.17	0.56 ± 0.17	0.417
PAPi	1.5 (1.0–2.4)	1.7 (1.0–2.4)	1.5 (1.0–2.7)	0.884

*Hypertension, CRT: 41 missing values; Wedge pressure: 65 missing

BMI: Body Mass index; CPO: cardiac Power Output; CRF: history of Chronic Renal Failure; CVP: Central Venous Pressure; DAP: Diastolic Arterial Pressure; MAP: Mean Arterial Pressure; mPAP: mean Pulmonary Artery Pressure; PAPi; Pulmonary Arterial Pulsatility index; SAP: Systolic Arterial Pressure.

### 3.3 Prediction model

**Fig. 2:**
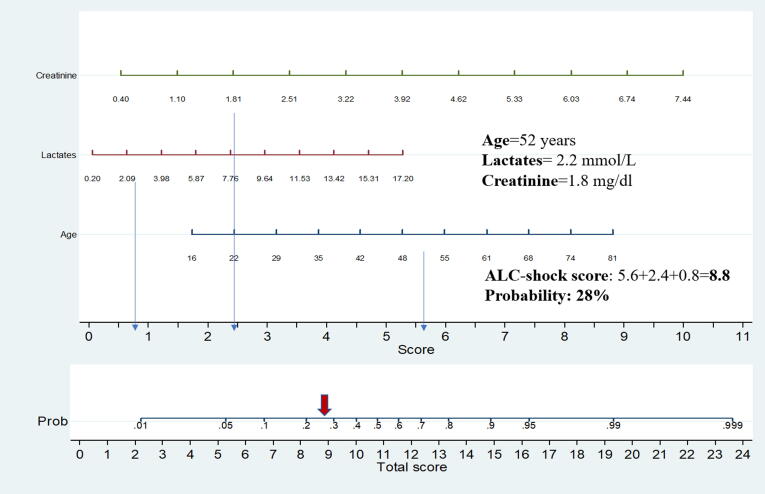
Title: Nomogram for predicting the probability of 28-day mortality. Caption: a logistic regression nomogram was obtained plotting all possible points for each variable, getting a costant, and transforming the results into a probability of event. For each variable, longer is the line, more important is the predictive value of the variable.

Age, serum creatinine and arterial lactates were identified as risk factors for 28-day mortality. The estimated ORs were 1.06 (95% CI = 1.01–1.108) for 1-year increase in age, 2.06 (95% CI 1.14–3.72) for 1-point increase in serum creatinine, and 1.18 (95% CI = 1.02–1.36) for 1-point increase in arterial lactates, respectively. Table 3 reports the ORs associated with one unit increment of each predictor, and the ORs associated with one standard deviation increment of each predictor. The probability of death at 28 days can be calculated based on the model coefficients. For example, the predicted probability for a 50 years old patient with creatinine 2 and lactate 3 = exp(-5.788832 + Lactates*0.1650827 + creatinine*0.7220503 + age*0.0584313), where −5.788832 is the intercept and the other coefficients (0.16 50827, 0.7220503, 0.0584313) are log transformed ORs. A nomogram was developed as a graphical tool using Stata software. In the above example, the total score is 0.28 in the nomogram that corresponds to a predicted probability of 28%. (Fig. 2).

**Table 3:** Results of the multivariable regression model showing the association between age, baseline creatinine and lactates and 28-day mortality.

Predictor	OR (95% CI)^1^	OR (95%)^2^	p Value	Predictive Information*
Age	1.06 (1.01–1.11)	1.10 (1.02–1.18)	0.010	37%
Creatinine	2.06 (1.14–3.72)	1.08 (1.01–1.155)	0.017	33%
Lactates	1.18 (1.02–1.36)	1.06 (1.01–1.123)	0.022	30%

1 OR associated with an increment of one unit of each predictor

2 OR associated with an increment of one standard deviation of each predictor

* calculated as percent of total chi square of the predictive model

### 3.4 Model calibration

**Fig. 3:**
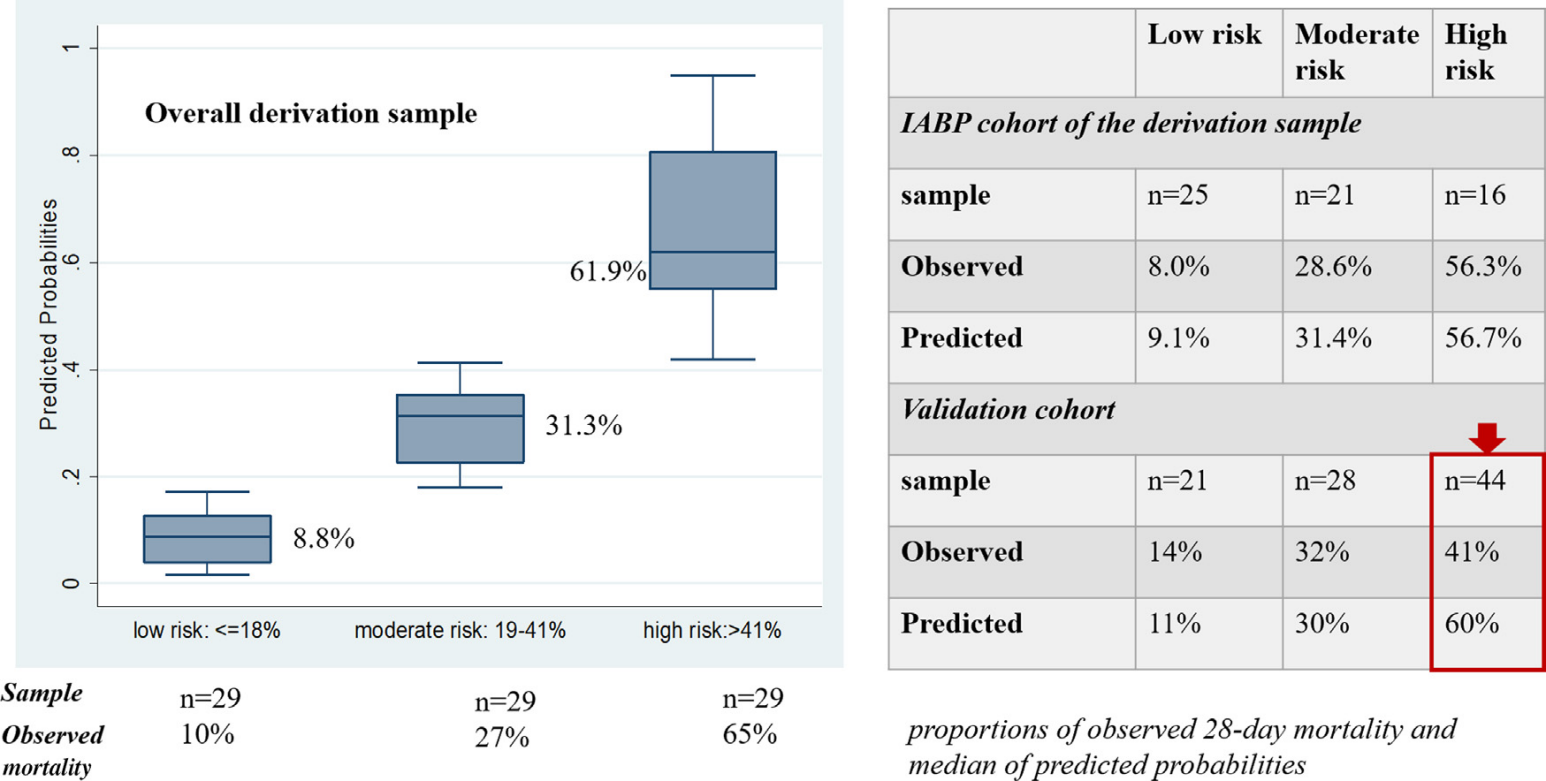
Title: Observed 28-day mortality stratified according to tertiles of predicted probabilities. Caption: Predicted probabilities were computed from the logistic model performed in the derivation cohort; these probabilities were divided in tertiles. For each sample (full derivation cohort; derivation cohort including only patients who had implanted IABP and validation cohort), the observed rates of 28-day mortality were plotted according to the above described tertiles of predicted probabilities. The ALC-shock score showed a good calibration in the original cohort, overestimating the risk in the third tertile for patients included in the validation population.

Fig. 3 shows the median of the predicted probabilities plotted against the proportions of observed 28-day mortality stratified according to tertiles of predicted probabilities. The risk of mortality was well predicted in the derivation cohort (for both the entire cohort and similarly in the subgroup of patients who were treated with IABP), whereas, in the validation cohort, it was well predicted in patients at low and moderate risk but was overestimated in the high-risk group.

### 3.5 Score validation

The c-statistic of the model was 0.82 (95% CI = 0.73–0.91) in the derivation sample as whole, and 0.80 (95% CI 0.66–0.92) when only patients receiving IABP were included. In the internal validation, the AUC value was comparable (0.80, 95% CI = 0.67–0.88), while in the external validation it decreased to 0.66 (95% CI = 0.54–0.78).

The prediction model was further evaluated by incorporating clinical outcome throughout the graphical display, in order to assess the net effect for 100 patients. Using a 40% threshold for 28-day mortality (i.e., all patients above this threshold were considered at high risk), the ALC shock score would classify correctly 21 events, while 12 classified at high risk would not die within 28 days from admission in ICU in a population of 100 patients with a 34% risk of 28-day mortality. Specifically, the choice of a 40% threshold results in a net gain of 13 patients (i.e., 21–0.66*12). The calculation of the net benefit was performed according to the risk threshold theory, where the gain of a patient correctly classified above the threshold is set as equal to one and the damage of a patient incorrectly classified below the threshold is equal to threshold/(1-threshold). This computation summarizes the utility of the model using one number Fig. 1 (***Appendix***).

### 3.6 Comparison between the ALC-shock score and the Cardshock score

**Fig. 4:**
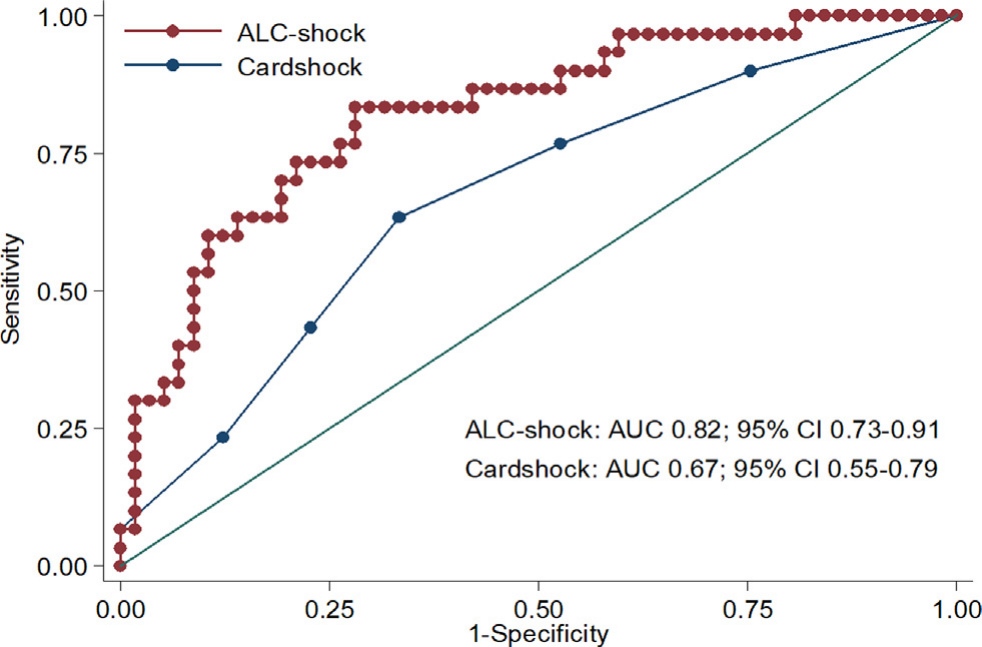
Title: ROC analysis of model-predicted rates of 28-day mortality and actual rates in the derivation cohort. Caption: Discrimination obtained using the receiver-operating curve (ROC) analysis was compared between the ALC-shock score and the Cardshock score. Area under the Curve (AUC) and 95% CI (confidence interval) are reported.

**Fig. 5:**
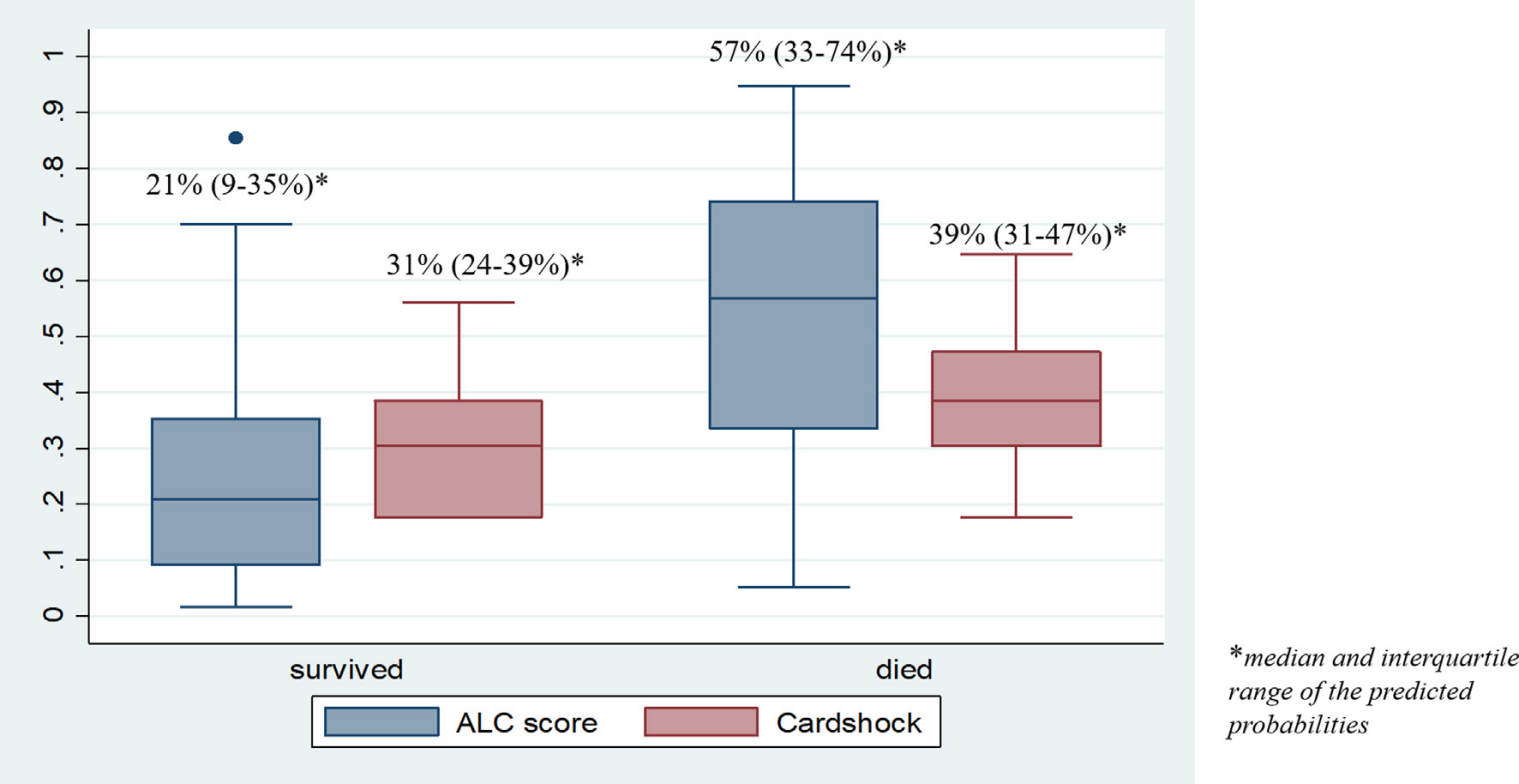
Title: Predicted probabilities of 28-day mortality of the ALC-shock versus the Cardshock score. Caption: For patients who survived and died, median and interquartile range of the predicted probabilities are reported for the ALC-shock score and the Cardshock score. The ALC-shock score produces lower predicted probabilities for low-risk patients (patients who survived) and higher predicted probabilities for high-risk patients (patients who died), compared to the Cardshock score, showing a better calibration.

Compared to the ALC-shock score, the Cardshock score showed a poorer discrimination (AUC 0.67; 95% CI 0.55–0.79; p = 0.009) (Fig. 4) and calibration (Fig. 5) when applied to the derivation cohort.

## 4 Discussion

Herein, we report on outcomes of a CS patient population limited exclusively to ADHF in two tertiary care centers that have access to heart replacement therapy. The main finding of this study is that a simple risk score which includes age, serum creatinine and arterial lactates may adequately predict 28-day mortality in ADHF-CS patients. This new, relatively simple score performed well when compared to a score incorporating more clinical factors which was derived from a heterogenous population of cardiogenic shock patients [13].

Clinical decision-making is challenging in CS patients due to the complexity of the metabolic, hemodynamic and inflammatory pathways that get activated once a low output state develops [3]. Moreover, the evolution in the epidemiology of CS that occurred in the last decades require further attention [6]: data from the collaborative research network of the American Heart Association identified a shift in CS etiology from AMI to ADHF [3]. These two scenarios differ from a pathophysiological and hemodynamic standpoint, [28] and this difference may explain why treatments that are ineffective in the setting of AMI (e.g., IABP) might be beneficial in patients with CS-ADHF.

Use of IABP appears particularly appealing since the risks of the procedure are low and the increase of cardiac output, although limited, can be sufficient to meaningfully improve tissue perfusion in patients with chronic advanced HF where adaptations to a chronically reduced cardiac output have already taken place. In addition, differently from AMI, the beneficial effects of CS management in ADHF are not confounded by results of concurrent reperfusion therapy.

Upon review of the CS literature, three variables were consistently identified as outcome predictors: age, renal function, and lactates [10], [13], [25], [28], [30] Ageing and renal failure are associated with poor outcomes in several cardiac and non-cardiac disease states [31], [32], [33]. Although these characteristics may eventually represent contraindications to HT or LVAD, they can also be regarded as early indicators for the need of heart replacement therapy, before deterioration of end-organ function becomes irreversible. For example, elevated arterial lactate is an early marker of that metabolic dysregulation, which leads to multiorgan failure [3]. Therefore, in patients with acutely decompensated chronic heart failure, these three variables reflect constitutive and contingent factors strongly related to prognosis.

Importantly, the performance of our score was somewhat reduced when applied to an external validation cohort. However, the derivation and validation population differed with respect to age and risk profile. Furthermore, every patient in the validation cohort was treated with an IABP, suggesting a potential impact of this therapy on outcomes. Importantly, when we compared actual and predicted mortality, the ALC-shock score was well-calibrated for all patients at low and intermediate risk including those included in the external validation cohort.

Recent data have focused on the prognostic role of hemodynamic indexes, such as cardiac power output/index (CPO/CPI) and pulmonary artery pulsatility index (PAPi), in patients with ADHF-CS [25], [30]. However, the insertion of a pulmonary artery catheter (PAC) is not routinely performed upon admission in every center, and, for this reason, baseline hemodynamic data may not be readily available. Several studies have recently shown the prognostic benefit associated with the use of PAC [34], [35]. Nevertheless, the same studies have also shown a marked decline of its use over the decades, and have attributed the improved outcome linked to its use to improvements in patient selection. Accordingly, a scientific statement from the American Heart Association recommends the use of PAC only in cases of diagnostic or CS management uncertainty, or in patients who are unresponsive to the initial therapy [36]. Likewise, a recent position statement from the Heart Failure Association of the European Society of Cardiology states that routine use of PAC remains contentious [37].

Moreover, patients with chronic AHF may have adapted to chronically abnormal hemodynamic profile with normal or near normal end-organ function, lactate and minimal symptoms. Thus, models that purely rely on hemodynamics parameters might be limited with respect to their prognostic ability and broader applicability. Indeed, a **major strength** of this report is the description of a simple, reliable and widely applicable prediction model among ADHF-CS patients, who represent a growing percentage of the overall CS population. This tool can be used at the bedside, upon admission to the ICU, for early prognostication and identification of those CS patients who may benefit from early use of mechanical circulatory support as bridge for durable surgical solutions such as HT or LVAD.

Our study has **several limitations**. First, we did not compare our score to others that included hemodynamic data [25]. However, as previously discussed, hemodynamic assessment with PAC is not routinely performed, limiting the widespread applicability of these scores. Second, as noted above, the model had diminished performance in the validation cohort, particularly among the highest risk profile. Differences in characteristics of the validation cohort, such as older age, higher wedge pressures and lower blood pressure, when compared to the derivation cohort, may explain these findings. However, both the Society for Cardiovascular Angiography and Intervention (SCAI) [38] and the ESC 2020 [37] have recognized how challenging is the trade-off between hypotension (often “relative” in the beginning states of CS) and hypoperfusion, and relied on the second one as a tool to promptly identify those patients at higher risk of death. Moreover, ADHF-CS patients have a more pronounced “congestive” profile than the AMI-related cardiogenic shock [37]. Thus, a different profile of our validation cohort may add to the generalizability of our results. Third, it is important to note that intermediate and long-term survival often depends on the patient’s candidacy for heart replacement therapies and the criteria for such therapies often differ among centers including the two that contributed to this analysis. Fourth, differently from other reports, [25] we considered LVAD/HT as neither a covariate nor an endpoint since our work focused on baseline prediction of 28-day mortality. Moreover, the high frequency of this treatment (around 2/3 of the patients) would have limited the usefulness of our prediction model.

Fifth, rounded coefficients in a numeric score could be easier to interpret than a nomogram. However, nomograms have been widely used across several diseases for prognostication, since they carry the important advantage of an easy estimation of probability of death using variables the relative importance of which is clear at a glance (i.e., a longer line corresponds to a more important variable) [39].

Lastly, we did not consider as predictors BNP or NT-proBNP levels, which have shown to be robust predictors of outcome in this acute setting [40]. However, we could not include this data since this biomarker was not systematically measured at admission in our retrospective cohorts.

## 5 Conclusions

Cardiogenic shock remains a deadly scenario where little is known about the management of patients with ADHF. In this setting congestion is the often the primary marker of decompensation and a primary target of treatment. Thus, dedicated stratification tools for this unique subgroup of CS patients are needed. Our results indicate that short-term mortality of patients with ADHF-CS can be adequately predicted upon admission based on patient’s age, serum lactate and serum creatinine (ALC-shock score). This stratification tool is easy to use and may help clinicians with in their everyday practice by identifying, early on, a high-risk cohort that might eventually benefit from more aggressive treatment strategies.

***Disclosures:*** Dr. Garan is supported by National Institutes of Health Grant No. KL2TR001874 and has received honoraria from Abiomed. Dr. Colombo reports institutional grant support from Abbott Vascular. Dr. Morici reports institutional grant support from Getinge Global and lecture fees Pfizer/Bristol-Myers Squibb. None of the listed entities has had any involvement with the development of the manuscript. All other authors have reported that they have no relationships relevant to the contents of this paper to disclose.
