# Supplementary figures and images for: Predicting survival in patients with acute decompensated heart failure complicated by cardiogenic shock
Source: Int J Cardiol Heart Vasc. 2021 Jun 4;34:100809. doi: 10.1016/j.ijcha.2021.100809 (PMC8188054; doi:10.1016/j.ijcha.2021.100809)

**Appendix Figure 1. Decision curve analysis. Net benefit of using a model to predict 28-day mortality**


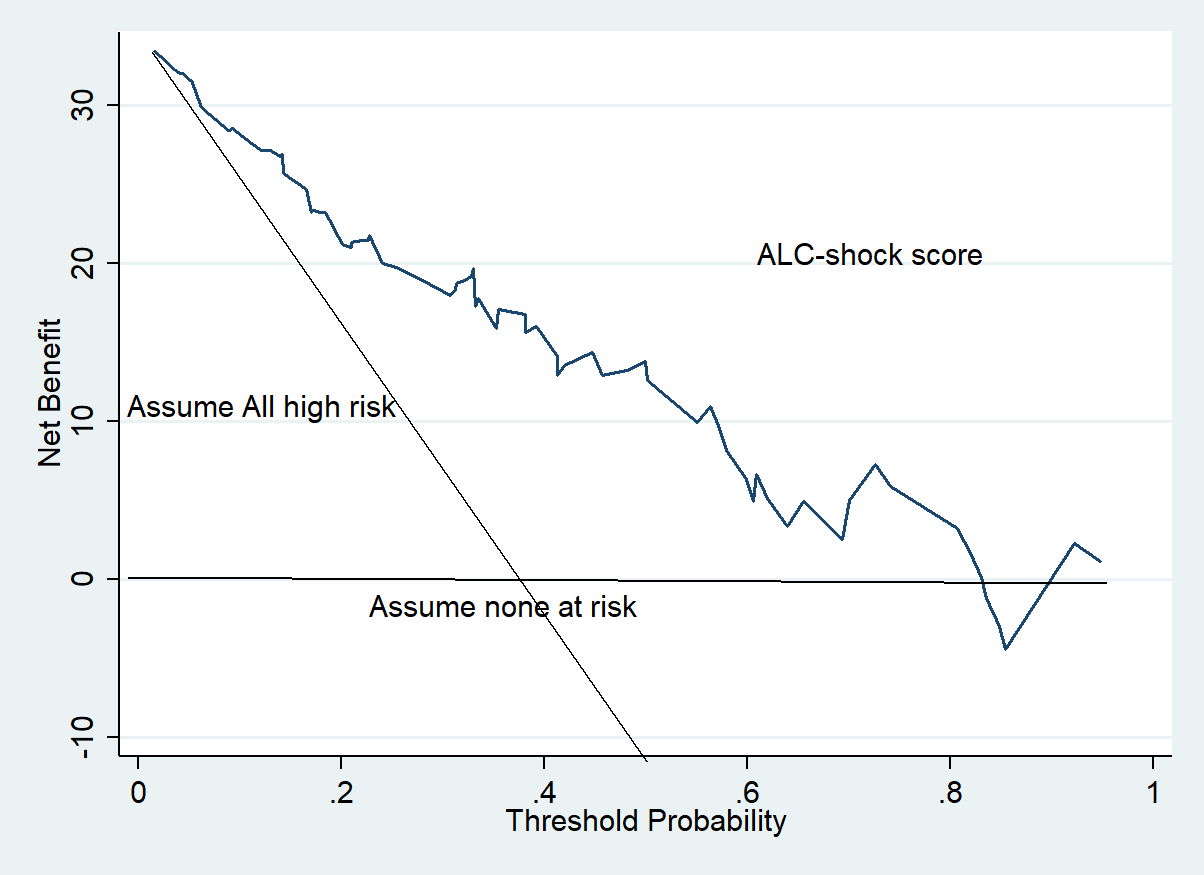

Supplement: Supplementary data 1 [file mmc1.docx]
